# P-2118. False-positive Aspergillus Galactomannan Test in Multiple Myeloma

**DOI:** 10.1093/ofid/ofae631.2274

**Published:** 2025-01-29

**Authors:** Shingen Nakamura, Yusaku Maeda, Ryohei Sumitani, Masahiro Oura, Kimiko Sogabe, Hikaru Yagi, Shiro Fujii, Takeshi Harada, Hirokazu Miki

**Affiliations:** Department of Community Medicine and Medical Science, Tokushima University Graduate School of Biomedical Sciences, Tokushima, Japan, tokushima, Tokushima, Japan; Department of Hematology, Endocrinology and Metabolism, Tokushima University Graduate School of Biomedical Sciences, Tokushima, Japan, Tokushima, Tokushima, Japan; Department of Hematology, Endocrinology and Metabolism, Tokushima University Graduate School of Biomedical Sciences, Tokushima, Japan, Tokushima, Tokushima, Japan; Department of Hematology, Endocrinology and Metabolism, Tokushima University Graduate School of Biomedical Sciences, Tokushima, Japan, Tokushima, Tokushima, Japan; Department of Hematology, Endocrinology and Metabolism, Tokushima University Graduate School of Biomedical Sciences, Tokushima, Japan, Tokushima, Tokushima, Japan; Department of Hematology, Endocrinology and Metabolism, Tokushima University Graduate School of Biomedical Sciences, Tokushima, Japan, Tokushima, Tokushima, Japan; Department of Hematology, Endocrinology and Metabolism, Tokushima University Graduate School of Biomedical Sciences, Tokushima, Japan, Tokushima, Tokushima, Japan; Department of Hematology, Endocrinology and Metabolism, Tokushima University Graduate School of Biomedical Sciences, Tokushima, Japan, Tokushima, Tokushima, Japan; Division of Transfusion Medicine and Cell Therapy, Tokushima University Hospital, Tokushima, Japan, Tokushima, Tokushima, Japan

## Abstract

**Background:**

Invasive pulmonary aspergillosis (IA) is a common infectious disease in patients with hematological diseases. The prevention, early detection, and establishment of treatment strategies for IA are important clinical issues. One of the mycological tests for IA is the serum galactomannan antigen (GM), which is included in the European Organization for Research and Treatment of Cancer-Invasive Fungal Infections Cooperative Group/National Institute of Allergy and Infectious Diseases Mycosis Study Group (EORTC/MSG) mycological criteria and is widely used because of its high sensitivity and specificity. However, whether this method is useful for all diseases and situations is unclear.

**Methods:**

We retrospectively analyzed the results of all GM tests performed in our department between April 2003 and January 2012.

**Results:**

Of 330 cases and 2155 samples, 540 (25%) were positive (0.5≦). The positivity rates for underlying diseases were 10.1%, 6.6%, 4.8%, 20%, 21.2%, and 61.3% for acute myeloid leukemia, acute lymphoblastic leukemia, chronic myelogenous leukemia, myelodysplastic syndrome, lymphoma, and multiple myeloma, respectively, with the highest positivity rate for myeloma. By type, IgG, IgA, Bence Jones protein, and IgD were 71.7%, 33.3%, 57.1%, and 34.6%, respectively, and the positivity rate of IgG type was high. All 18 IgG-positive cases at the time of diagnosis of MM were false positives according to the 2019 EORTC/MSG criteria. There was no direct correlation between the IgG and GM values. Of the 18 false-positive cases, 12 patients received antifungal prophylaxis, two of whom developed IA during treatment with steroids, and five who did not receive prophylaxis did not develop IA. When the Aspergillus antigen was measured using the serum from which IgG-type monoclonal protein in some patients with IgG-type myeloma was purified and extracted using protein G, the Aspergillus antigen values decreased.

**Conclusion:**

Although it is possible that the GM test for myeloma captures subclinical IA, the GM antigen test may be prone to false positives in IgG-type myeloma, and the results should be interpreted with caution.

**Disclosures:**

All Authors: No reported disclosures

